# The Optimal Surgical Approach to Intradural Spinal Tumors: Laminectomy or Hemilaminectomy?

**DOI:** 10.7759/cureus.7084

**Published:** 2020-02-23

**Authors:** Amir Goodarzi, Jared Clouse, Tatiana Capizzano, Kee D Kim, Ripul Panchal

**Affiliations:** 1 Neurological Surgery, University of California Davis Medical Center, Sacramento, USA; 2 Neurological Surgery, University of California Davis School of Medicine, Sacramento, USA; 3 Neurological Surgery, American Neurospine Institute, Plano, USA

**Keywords:** laminectomy, hemilaminectomy, intradural, spinal tumor, intramedullary, extramedullary

## Abstract

Objective

Traditionally, laminectomy has been the preferred surgical approach for the resection of intradural spinal tumors. Recent trends towards minimally invasive techniques have generated interest in hemilaminectomy as an effective alternative surgical approach to resect spinal tumors. However, it remains unclear if the potential benefits of hemilaminectomies, used in other routine spinal procedures, apply to intradural spinal tumors. This report presents a six-year single institutional analysis of open resection of intradural tumors using laminectomies as compared to hemilaminectomies.

Methods

A single institution, multisurgeon, retrospective review of 52 patients undergoing resection of intradural spinal tumors over a six-year period was performed. Estimated blood loss, operative time, post-operative complications, length of stay, and post-operative clinical spinal instability were analyzed and compared between the two surgical techniques.

Results

The mean follow-up was 34 and 20 months for the laminectomy and hemilaminectomy groups, respectively. There was no statistically significant difference in operative times between the two groups (hemilaminectomy: 250.13±76.44 minutes, laminectomy: 244.49±92.85 minutes; p=0.43). Similarly, there was no difference in overall estimated blood loss (hemilaminectomy: 125±74 cc, laminectomy: 256.05±320.8 cc; p=0.27) or mean hospital length of stay (hemilaminectomy: 4.00±2.12 days, laminectomy: 5.26±3.0 days; p=0.60). No patient in either surgical group had post-operative evidence of clinical spinal instability.

Conclusion

Hemilaminectomy is a viable approach for the resection of intradural spinal tumors, with similar rates of post-operative complications to laminectomy when using an open surgical approach. The laminectomy allows for bilateral exposure of the entire spinal canal and neural foramina; and continues to be the preferred method for resection of large tumors with complex morphology.

## Introduction

Intradural spinal cord tumors, although frequently of benign histopathologic origins, can lead to compressive myelopathy and radiculopathy due to rigid architecture of the spinal canal and neural foramina [1]. When neurological deficits are present, including myelopathy and radiculopathy, surgery is considered to prevent further neurological decline.

Historically, the posterior laminectomy has been the favored approach for approaching intradural spinal tumors [1,2]. In 1829, Alban Smith described the first successful posterior laminectomy to access the spinal canal for decompression of the spinal cord in the setting of trauma [3]. In 1887, Sir Victor Horsley was the first to adopt this approach for the removal of an intradural myxoma [3,4]. Since that time, numerous variations of the posterior laminectomy have been implemented for the resection of a variety of spinal pathologies. However, recent clinical and radiographic studies have demonstrated the potential for post-operative kyphosis, instability, and increased pain associated with laminectomies. The pediatric population and patients with cervical intradural tumors are at the highest risk for these complications [5-9]. Previously published results suggest that these complications are secondary to disruption of the posterior spinal tension band, including the paraspinal musculature, the spinous processes, and the supraspinous and interspinous ligaments, and the zygapophyseal joints (spinal facets) [5-10].

Unilateral hemilaminectomy for resection of intradural tumors was first proposed in 1989 and 1991 by Chiou and Yasargil [3,5]. The hemilaminectomy technique minimizes the bony removal and allows for preservation of the spinous process, the contralateral zygapophyseal joints, and the contralateral paraspinal musculature, which could potentially reduce the risk of post-operative instability, kyphosis, and pain [3,5,8]. Recent data have demonstrated positive clinical outcomes with respect to blood loss, complications, length of hospital stay, operative time, and most importantly post-operative instability when utilizing a hemilaminectomy approach [11-18]. However, it remains unclear if these benefits are due to the reduced bony resection or the utilization of minimally invasive surgical (MIS) techniques compared to open surgery.

Given the recent trend towards minimally invasive approaches, we set out to study the significance of the amount of bony resection (laminectomy versus hemilaminectomy) as an independent predictor of positive clinical outcomes for the resection of intradural spinal tumors, irrespective of the surgical technique used to approach the spine (minimally invasive or open approach). This question is especially pertinent in the setting of intradural tumors where exposure of the tumor is critical in achieving maximal safe resection without compromising neurological function.

## Materials and methods

A retrospective review of all patients with intradural spinal pathology from 2009 to 2015 was performed. Four surgeons were involved in spinal operations during this interval. Patients were excluded from the study if the tumor was not located within the thecal sac or if the surgical approach used was not a laminectomy or hemilaminectomy. No other exclusion criteria were used in patient selection for this study. Analyzed variables include pre-operative tumor size, residual tumor post-operatively, estimated blood loss, operative time, length of hospital stay, post-operative complications, and post-operative instability. Complications included intraoperative blood loss, length of hospital stay, need for reoperation, new-onset weakness, or sensory deficit. Post-operative stability was assessed by examining the need for repeat surgery, and clinically significant symptoms of pain, weakness, and sensory deficits. Imaging was unable to be used to assess for spinal stability due to insufficient available data. The institutional review board and the cancer center scientific review committee at University of California Davis Medical Center approved this study (Number 810111).

Tumor size was determined by measuring the lesion size on pre-operative magnetic resonance imaging (MRI) scans. Cranial-caudal (C-C) measurements were obtained from sagittal sequences, whereas axial sequences were used to determine anterior-posterior (A-P) and medial-lateral (M-L) measurements. C-C, A-P, and M-L measurements were multiplied together and divided by 2 to calculate the three-dimensional volume of each tumor. Residual tumor size was determined by reviewing the immediate post-operative MRI, utilizing the above-mentioned method.

Statistical analysis software was used to compare the laminectomy and hemilaminectomy groups and to calculate mean, standard deviations, two-sided Wilcoxon rank-sum test, and two-sided t-tests for the variables analyzed in this study. Statistical significance was determined for all analyzed variables with a p-value <0.05.

Surgical technique

The decision to perform hemilaminectomy versus laminectomy was primarily based on tumor size. Smaller tumors were more amenable to resection with a hemilaminectomy, whereas larger tumors required laminectomy for adequate resection. No neuromonitoring was used during any of the cases included in this study.

Laminectomy 

After using intraoperative x-rays to localize the spinal level of interest, a midline posterior incision was made. An avascular tissue plane was identified in the mid-sagittal plane, and the dissection was carried down to the spinous process using monopolar cautery. Subperiosteal dissection was used to dissect the paraspinal musculature away from the lamina, allowing for removal of the lamina, ligamentum flavum, and exposure of the dura and neural elements. Extreme caution was taken to preserve the zygapophyseal joints bilaterally. The dura was incised and tacked up using 4-0 nylon sutures. The tumor was then resected using a combination of bipolar cautery, sharp, and blunt dissection. Primary dural closure was obtained after tumor removal, followed by anatomic realignment of the paraspinal musculature, fascia, dermis, and epidermis.

Hemilaminectomy 

Once the desired level of pathology was localized, a paramedian incision was made and monopolar cautery was used to dissect down to the spinous process, taking great care to preserve the supraspinous and interspinous ligaments. A subperiosteal dissection was carried down the side of the lamina and laterally to expose the pars interarticularis and zygapophyseal joint on the side of the lesion. The lamina was then drilled down using a matchstick drill starting at the midline and extending to just medial to the zygapophyseal joint. Care was taken to ensure the exposure was adequate for intradural resection. In cases where lateral pathology was encountered a minimal medial facetectomy was performed, removing no more than one-third of the facet joint. The tumor was then resected using a combination of bipolar cautery, sharp, and blunt dissection. Primary dural closure was obtained after tumor removal, followed but anatomic realignment of the paraspinal musculature, fascia, dermis, and epidermis.

## Results

Demographics

A total of 52 patients were included in this study with nine individuals undergoing hemilaminectomy and 43 individuals undergoing complete laminectomy. The hemilaminectomy group comprised 22% male with an average age of 42 years old (standard deviation [SD], 19 years; range, 10-64 years), whereas the laminectomy group comprised 49% male with a mean age of 52 years (SD, 14 yrs; range, 20-70 years). All patients who underwent hemilaminectomy had medical comorbidities. whereas only 70% of the laminectomy patients had similar comorbid conditions. Comorbid conditions included history of cancer, obesity, heart disease, thyroid disease, and history of tobacco use. The mean follow-up time was 34 months (range, 1-92 months) in the laminectomy group and 20 months (range, 2-84 months) in the hemilaminectomy group.

Location of tumors

In the hemilaminectomy group, tumors were located primarily in the cervical region with five patients (55%) having tumors in this region. The tumors in the other four patients were located in the cervicothoracic, thoracic, thoracolumbar, or lumbar regions, respectively. Patients who underwent laminectomy more commonly had tumors in the thoracic (40%), cervical (21%), and lumbar (19%) spine regions. Tumors in junctional regions (cervicothoracic, 2%; thoracolumbar, 12%) and the sacrum (5%) were less common in this group (Table 1).

**Table 1:** Distribution of intradural tumors based on location and surgical route utilized.

	Hemilaminectomy		Laminectomy	
Location	n	%	n	%
Cervical	5	56	9	21
Cervicothoracic	1	11	1	2
Thoracic	1	11	17	40
Thoracolumbar	1	11	5	12
Lumbar	1	11	8	19
Lumbosacral	0	0	1	2
Sacral	0	0	2	5

Tumor types

Meningioma was the most frequent tumor pathology in both cohorts (35% laminectomy group, 33% hemilaminectomy group). A total of 33% of patients in the hemilaminectomy group and 23% of laminectomy patients had schwannomas. Ependymomas were more common in the laminectomy group (23% versus 11%). Other tumor pathology in the laminectomy group included paraganglioma (5%), neurofibroma (2%), lymphoma (2%), hemangioblastoma (2%), hemangioma (2%), mature teratoma (2%), and pineoblastoma (2%). Additionally, in the hemilaminectomy cohort a single occurrence of both hemangioma and neurofibroma was observed (Table 2).

**Table 2:** Distribution of tumors resected by surgical approach and tumor pathology.

Laminectomy Tumor Type	n	%	Hemilaminectomy Tumor Type	n	%
Meningioma	15	35	Meningioma	3	33
Ependymoma	10	23	Ependymoma	1	11
Schwannoma	10	23	Schwannoma	3	33
Paraganglioma	2	5	Paraganglioma	0	0
Neurofibroma	1	2	Neurofibroma	1	11
Lymphoma	1	2	Lymphoma	0	0
Hemangioblastoma	1	2	Hemangioblastoma	0	0
Hemangioma	1	2	Hemangioma	1	11
Mature Teratoma	1	2	Teratoma	0	0
Pineoblastoma	1	2	Pineoblastoma	0	0
Total	43	-	Total	9	-

Tumor size

The mean pre-operative tumor size in the hemilaminectomy group was 1.25±0.86 cm^3^ (range, 0.39-2.9 cm^3^). In contrast, the laminectomy group had a mean size of 7.02±24.20 cm^3^ (range, 0.06-146.15 cm^3^; p=0.47). Post-operatively, all patients in the hemilaminectomy group had a gross total resection with no residual tumor on MRI. In contrast, only 79% of patients who underwent laminectomy had a gross total resection, with a mean residual tumor size of 2.89±5.94 cm^3^ and a maximum residual tumor size of 18.68 cm^3^ (Table 3).

**Table 3:** Mean tumor size and extent of resection based on surgical approach.

Surgical Approach	Mean Tumor Size (cm^3^)	SD	Gross Total Resection %
Hemilaminectomy	1.25	±0.86	100
Laminectomy	7.02	±24.20	79

Complications

There was no significant difference in length of operation between the two approaches (hemilaminectomy: 250.1±76.4 minutes, laminectomy: 244.5±92.9 minutes; p=0.43). Similarly, there was no difference in estimated blood loss (hemilaminectomy: 125±74 ml, laminectomy: 256±321 ml; p=0.27) or hospital length of stay (hemilaminectomy: 4.00±2.1 days [range, 2-9 days], laminectomy: 5.3±3.0 days [range, 2-15 days]; p=0.60) (Table 4). 

**Table 4:** Mean estimated blood loss and post-operative hospital length of stay categorized by surgical approach.

	n	Mean Estimated Blood Loss (cc)	SD	Mean Post-op Hospital Length of Stay (Days)	SD
Hemilaminectomy	9	125	±74.40	4.00	±2.12
Laminectomy	43	256.05	±320.8	5.26	±3.00

There were no mortalities within the hemilaminectomy group. A single patient (11%) experienced new-onset foot drop post-operatively after a hemilaminectomy. While another patient complained of new-onset lower extremity dysesthesias (11%). During the follow-up period, there were three mortalities within the laminectomy cohort, none of which were related to the operation. Two patients succumbed to metastatic cancer, and another patient suffered heart failure after contracting pneumonia eight months after surgery. Among patients who underwent laminectomy, acute post-operative hemiparesis was seen in one patient (2%). Another patient complained of post-operative hand and arm weakness (2%), which required emergent return to the operating room for untethering of adhesions. While one additional patient was found to have post-operative cauda equina syndrome secondary to a pseudomeningocele and seroma, which required return to operating room for evacuation (2%). 

Using clinically significant symptoms of pain, weakness, sensory deficit, and the need for repeat surgery as surrogates for post-operative instability, during follow-up period there was no evidence of instability in either group.

## Discussion

In 1829, the first attempt of laminectomy provided the framework for future surgeons to address spinal tumor pathologies [4,5]. Although many new surgical corridors have been conceived since that time, laminectomy remains one of the primary approaches for removing intradural pathology. However, long-term outcomes on patients who underwent laminectomies, particularly in vulnerable populations such as children, have raised concerns of post-operative instability and kyphosis [7,8,10,19].

Recent trends towards MIS techniques and cost-effective utilization of limited hospital resources have introduced questions regarding which surgical approaches should be used to access spinal pathologies. Moreover, multiple studies have confirmed the potential for reduced estimated blood loss, and lower length of hospital stays with MIS techniques [1,5,8,14-17,20]. These favorable results are typically attributed to the minimal disruption of the posterior tension band with little mention of the effects of the extent of bony resection. Our objective in this study was to assess the effects of the bony resection, laminectomy versus hemilaminectomy, on the ability to achieve gross total resection, and its role in post-operative recovery when using an open surgical approach. By using an identical open surgical approach in the two groups, we hoped to tease out any potential benefits that are directly attributable to the extent of bony resection without the confounding benefits that are related to MIS techniques.

While not statistically significant, there was a trend towards a reduction in blood loss in the hemilaminectomy group compared to the laminectomy group. This result was in contrast to published reports which showed a reduction in blood loss when utilizing MIS techniques for hemilaminectomy [14,21]. This finding suggests that the level of bony resection is not the major source of blood loss in these cases, and that the approach to the spine, MIS techniques versus open, may play a larger role in reducing total blood loss. Additionally, there was no significant difference in operative times or in the length of hospital stays between the two approaches. In contrast, the literature on MIS techniques has described shorter operative times with hemilaminectomies [14,15]. Again, this may indicate that the approach used to access the spine is more critical in operative times and post-operative recovery times than the extent of bony resection, but this cannot be definitively stated from the small size presented here.

The difference in pre-operative tumor size between the groups was not statistically significant; however, the largest tumor volume resected in the laminectomy was 146.1 cm^3^ compared to 2.9 cm^3^ in the hemilaminectomy group. Gross total resection was achieved in 100% of the hemilaminectomy group compared to 79% in the laminectomy group. One implication of these findings may be that in cases of smaller tumor volumes (less than 3 cm^3^) hemilaminectomy is a viable option and provides adequate visibility and access for safe and complete resection, whereas larger tumors may require a laminectomy for safe and complete resection. Lee et al. noted similar results in a series of 26 patients who underwent successful resection of spinal schwannomas less than 16 mm in axial diameter regardless of foraminal or sagittal extension, utilizing hemilaminectomies [1]. Additionally, tumor histopathology and location are important considerations when selecting the appropriate approach. A large series of spinal tumors utilizing laminectomy and hemilaminectomy concluded that juxtamedullary tumors of benign pathology were ideal for hemilaminectomies, whereas malignant tumors with complex morphology required a full laminectomy for adequate exposure and resection [5].

Another critical variable to consider when choosing a surgical corridor to an intradural spinal tumor is preservation of spinal stability. It has been noted by previous authors that kyphosis and post-operative instability occur secondary to the disruption of posterior spinal tension band [6-11]. Unfortunately, at our institution routine imaging was not performed for laminectomies or hemilaminectomies, and thus there was no direct measure of instability in the two groups. However, we analyzed the rates of repeat surgical interventions required between the two groups as a surrogate for post-operative instability. During the follow-up period, no patients in either group required repeat surgery to address spinal instability. Thus, implicitly, we suggest that there was no difference in the rates of clinically relevant spinal instability between the two surgical approaches. Further studies with pre- and post-operative imaging and larger sample sizes would aid in elucidating the effects of hemilaminectomy versus laminectomy on spinal stability after resection of intradural spinal tumors. 

The findings presented here suggest that the extent of bony resection (laminectomy versus hemilaminectomy) during the resection of intradural tumors does not significantly affect post-operative complications. Furthermore, given that there were no significant differences between the length of operation, length of stay, and blood loss between the hemilaminectomy and laminectomy, we propose that the MIS approach is potentially the reason for faster recovery and reduced blood loss, and that the extent of bony removal is far less significant factor but additional studies are needed to substantiate this claim. Excellent results can be obtained utilizing a hemilaminectomy to resect small and laterally located intradural tumors via an open surgical approach, and that surgeon preference and level of comfort should be taken into consideration. As with any surgical procedure, patient selection in an important aspect of achieving desirable surgical outcomes, and thus it is our view that for patients with large tumors with complex morphology the laminectomy approach may provide optimal exposure compared to a hemilaminectomy, which is ideal for small tumors with lateral extension. In the hemilaminectomy group, a more aggressive facetectomy may be undertaken given that the contralateral joint is undisturbed and not exposed during the approach. This allows for exposure of lateral tumors without significantly compromising spinal stability.

Limitations

Limitations of this study include its retrospective nature, small sample size, unequal group sizes, and the inability to assess post-operative spinal stability with imaging. None of the results presented here reached statistical significance, which may be secondary to the small size of the groups examined. The groups had marked differences in size, location, and pathology of tumors resected making it difficult to make accurate determinations about differences between the groups. Future studies should include a larger sample size with matched patient cohorts in order to more accurately assess observed differences between the groups. Additionally, pre- and post-operative imaging should be obtained to assess radiographic spinal stability in any future studies. 

## Conclusions

Due to the small sample size and the disproportionate number of patients in each group, definitive conclusions are difficult to make in the data presented; however, hemilaminectomy is a viable alternative to the laminectomy for resection of small intradural tumors when using an open surgical approach. For patients with large tumors with complex morphology, the laminectomy approach may provide optimal exposure compared to a hemilaminectomy, which is ideal for small tumors with lateral extension. 
